# The biological screening of aromatic medicinal plant extracts for their potential *in vitro* bioactivity against factors associated with breast mastitis

**DOI:** 10.3389/fphar.2026.1893713

**Published:** 2026-09-01

**Authors:** Elizabeth Jean Wildy, Samantha Rae Loggenberg, Namrita Lall

**Affiliations:** Department of Plant and Soil Sciences, University of Pretoria, Pretoria, South Africa

**Keywords:** antibacterial, aromatic plants, breast mastitis, cytotoxicity, phytochemicals, *Staphylococcus aureus*, wound healing

## Abstract

Breast mastitis is an inflammatory condition caused by pathogenic infection, which is usually treatable with antibiotics, yet there is a lack of research focusing on plant-based alternatives. Aromatic plants are rich in secondary metabolites which potentially exhibit antibacterial and anti-inflammatory bioactivity. This study aimed to investigate ethanolic extracts of six aromatic plant species, namely,; *Allium schoenoprasum* L., *Allium* × *proliferum* (Moench) Schrad. Ex Willd., *Coleus amboinicus* Lour., *Eriocephalus africanus* L., *Pelargonium graveolens* L’Her., and *Salvia aurea* L., for their antibacterial activity against the breast mastitis-associated pathogen, *Staphylococcus aureus* (ATCC 25923), as well as their cytotoxicity and wound healing potential against human keratinocytes (HaCaT). Extracts with relatively high antibacterial activity, and low cytotoxicity, underwent gas chromatography-mass spectrometry (GC-MS) to identify potential bioactive compounds. *Coleus amboinicus, E. africanus*, *P. graveolens* displayed antibacterial activity against *S. aureus* ATCC 25923 (with minimum inhibitory concentrations (MIC) of 250 μg/mL for all extracts, and only *E. africanus* was found to have a minimum bactericidal concentration (MBC) at the tested concentrations (MBC of 1,000 μg/mL)*. Salvia aurea* was found to have the strongest antibacterial activity (MIC value of 15.625 μg/mL) and had an MBC of 250 μg/mL. Most extracts displayed minimal toxicity against HaCaT cells (IC_50_ > 1,000 μg/mL), excluding *S. aurea* (IC_50_ of 75.80 ± 4.58 μg/mL). *Allium* × *proliferum, C. amboinicus, and P. graveolens* displayed statistically significant wound healing activity, while *E. africanus* exhibited wound healing inhibition. Compounds such as camphor (identified in an abundance of 20.45% in *S. aurea*), and carvacrol (identified in an abundance of 5.08% in *C. amboinicus* and 6.63% in *P. graveolens*) were identified which may contribute to the observed bioactivity. It has been observed that the chosen aromatic plants may provide secondary metabolites that could potentially contribute to advances in breast mastitis treatment. Future prospects of this research include exploring aspects such as inflammatory signaling pathways or to isolate compounds such as camphor and carvacrol to determine whether these compounds are responsible for the observed bioactivity.

## 1 Introduction

Breast mastitis is an inflammatory condition which occurs in the breast and nipple tissue. Although it may also occur in non-lactating women, breast mastitis most commonly occurs in lactating women, and is usually observed during the first few weeks of the puerperium (the period after childbirth during which the reproductive organs return to their non-pregnant condition) and can be caused by bacterial pathogens, such as *Staphylococcus aureus*, or by milk stasis. Milk stasis is the primary cause and occurs when breast milk remains in the breast duct due to the infant being improperly attached during feeding, which causes a blockage (Pevzner and Dahan, 2020).

*Staphylococcus* has been identified as the predominant pathogenic genus in milk samples collected from women inflicted with mastitis, with *S. aureus* showing the highest causation rate (75 0%) (Angelopoulou et al., 2018). Mastitis caused by *S. aureus* can result in severe clinical symptoms beginning from infection, such as a high-grade fever (38.5 °C), burning pain, or engorgement (a swelling, and tightness resulting in an increase in size of the breast) (Baeza, 2016). These symptoms would most commonly arise in patients who contracted the infection from a methicillin-resistant *S. aureus* (MRSA) strain, which could potentially be, fatal (Li et al., 2021). The typical treatment regimen is antibiotics, which are prescribed if symptoms do not clear up after 24h. Antibiotics are often over-prescribed and unnecessary to treat breast mastitis caused by milk stasis which can lead to antibiotics being misused (Webster, 2017). Antibiotic misuse and overuse cause antimicrobial resistance by creating an evolutionary pressure which allows bacteria to adapt and survive, rendering the antibiotic of its ability to kill bacteria which becomes resistant (Ventola, 2015). Therefore, antibiotics are not always a viable option which gives reason to look at alternative treatments, such as medicinal plant products (Pignataro et al., 2020). Untreated breast mastitis can result in an abscess forming on the breast which needs to be surgically drained. Post-surgery breastfeeding mothers cannot continue breast feeding due to risk of infection as such a treatment which encourages wound healing would allow for a quicker return to breastfeeding (Feoktistova et al., 2021).

Aromatic plants have been used for centuries as natural medicines due to their high content in phenolic and bioactive compounds (Christaki et al., 2012). The characteristically strong scents given off by aromatic plants are essential oil compounds that are volatile at room temperature. These essential oils are a mixture of secondary metabolites that can act as defense for the plants, by deterring herbivory (by being released upon mechanical damage caused by herbivores), or to attract pollinators, and ensure zoochory (seed dispersal by animals) by consumption of seeds. Many studies have been done exploring the wide range of medicinal uses that derived essential oils possess, including antibacterial and anti-inflammatory properties which may aid in the search for novel drugs to treat mastitis (Samarth et al., 2017).

Six readily available aromatic plants were chosen for research in this study, namely, *Allium schoenoprasum* L., *Allium* × *proliferum* (Moench) Schrad. Ex Willd.*, Eriocephalus africanus* L., *Coleus amboinicus* Lour., *Pelargonium graveolens* L’Hér and *Salvia aurea* L. *Allium schoenoprasum* is a perennial bulb-forming herbaceous plant, characterized by edible, slender leaves. It is believed to originate from Siberia from which it spread across Asia, Europe, and North America. In East Asia, *A. schoenoprasum* is used to relieve cold, flu, and lung congestion as well as alleviate pain from sun burn and sore throat (Alam et al., 2023). *Allium* × *proliferum* (hybrid plant of *Allium cepa* L. and *Allium fistulosum* L.) is a perennial plant consisting of a cluster of bulblets where a normal onion would have a flower. It grows up to 1.5 m long and 20 cm broad, the leaves are cylindrical, hollow, and dark green with a pungent smell similar to that of onion. The plant is a garden crop most commonly planted in North America, Europe and Northeast Asia, yet is cultivated worldwide (Friesen and Klaas, 1998). It is traditionally used for culinary purposes; however, the species has been found to be useful in preventing oral infection and tooth decay, and the juice of the bulb is useful for treating wounds, insect stings, or fungal infections (Chevallier, 1996). *Eriocephalus africanus* is a hardy plant native to South Africa. The leaves are arranged in tufts around woody branches, they are small, and needle-shaped with minute silvery hairs covering the leaf, when crushed the leaves have a scent similar to that of Vicks™, the commonly known mentholated topical ointment (Salie et al., 1996). It is ingested orally to treat fever, swelling, and headaches (Scott et al., 2008). *Coleus amboinicus* is a perennial herbaceous plant with simple, thick leaves are that have a tapered tip, and covered in numerous glandular hairs, the flowers appear on a fluorescence-like structure with tiny purple flowers (Arumugam et al., 2016). The odour of *C. amboinicus* is described as refreshing and is similar to that of oregano. It is usually found growing in the tropics of Africa, Asia, and Australia (Arumugam et al., 2016; Kumar et al., 2020). *Coleus amboinicus* treats a broad range of ailments including constipation, fever, bronchitis, headache, and ear pain (Kumar et al., 2020). *Pelargonium graveolens* L’Hér is an erect shrub with a woody base, its leaves are deeply incised, velvety, lobed, and have an intense sweet and rose-like scent. *Pelargonium graveolens* has been used for centuries as a medicine in Africa. It is used to treat digestive issues, respiratory disease, and wounds. The leaves are pounded or made into a paste when used to treat wounds (Amel et al., 2022). *Salvia aurea* L. is an evergreen shrub with aromatic leaves, it is traditionally brewed as a tea and used to treat coughs, colds, and menstruation cramps (Ezema et al., 2023). The plants selected for this study (Table 1) have not been deeply evaluated for their antibacterial and wound healing potential, or the compounds have not been identified that are responsible for their bioactivity. No minimum inhibitory concentrations (MICs) against *S. aureus* have been previously reported for *A. × proliferum* and *S. aurea*.

**TABLE 1:** Literature review compiling the previously recorded minimum inhibitory concentration (MIC) against *Staphylococcus aureus* (µg/mL) of the plants chosen. No MIC values have been reported for *Allium × proliferum* and *Salvia aurea*.

Plant name	Type of extract tested	Minimum inhibitory concentration (MIC) against *Staphylococcus aureus* (µg/mL)	References
*Allium schoenoprasum* L	Dried whole plant ethanol extract	Displayed an MIC value 15 μg/mL against *S. aureus* ATCC unspecified	(Ghasemian et al., 2018)
	Dried whole plant aqueous extract	Displayed an MIC value 15 μg/mL against *S. aureus* ATCC unspecified, no cytotoxicity tested	
*Eriocephalus africanus* L	Dried root methanol extract	Displayed an MIC value of 10 mg/mL against *S. aureus* ATCC 25923	(Salie et al., 1996)
*Coleus amboinicus* (Lour.) Spreng	Fresh leaves ethanol extract	Displayed an MIC value of 9.3 mg/mL against *S. aureus* ATCC 25923	(Gurgel et al., 2009)
	Fresh leaves ethanol extract	Displayed an MIC value of 9.3 mg/mL against *S. aureus* ATCC 6538	
*Pelargonium graveolens* L'Hér	Fresh aerial parts of floral budding plant essential oil	Displayed an MIC value of 0.31–0.62 μg/mL against *S. aureus* ATCC 6538	(Boukhris et al., 2015)
	Fresh aerial parts of flowering plant essential oil	Displayed an MIC value of 0.62–1.25 μg/mL against *S. aureus* ATCC 6538	
	Fresh aerial parts of post-flowering plant essential oil	Displayed an MIC value of 0.62–1.25 μg/mL against *S. aureus* ATCC 6538	
	Fresh aerial parts of vegetative plant essential oil	Displayed an MIC value of 0.62–1.25 μg/mL against *S. aureus* ATCC 6538	

The aim of this study was to determine whether the ethanolic extracts of the six chosen aromatic plants may have potential for novel therapeutic which treat the factors associated with breast mastitis. This will be done by evaluating extracts of the plants for their antibacterial activity against the mastitis-associated bacteria, *S. aureus* (ATCC 25923), and potential cytotoxicity towards human skin cells, the human keratinocytes (HaCaT) cell line as well as the wound healing capability of the extracts against HaCaT. Furthermore, the aromatic compounds present in these extracts, which may potentially be responsible for the displayed bioactivity, were subsequently identified utilizing gas chromatography-mass spectrometry (GC-MS).

## 2 Materials and methods

### 2.1 Materials and reagents

The bacterial strain of *S. aureus* (ATCC 25923) was purchased from Anatech Analytical Technology (Johannesburg, South Africa). Reagents used for antibacterial assays, namely, Tryptic Soy (TS) Agar, TS Broth, and vancomycin were supplied by Sigma Chemicals Co. (St. Louis, Missouri, United States of America). The human keratinocyte (HaCaT) cell line was donated by the University of Cape Town (Cape Town, South Africa). Reagents used for cell culture maintenance, namely, Dulbecco’s Modified Eagle Medium (DMEM), foetal bovine serum (FBS), phosphate buffer system (PBS), penicillin, streptomycin, and amphotericin B were obtained from ThermoFisher Scientific (Johannesburg, South Africa). Cell culture consumables, which include sterile cell culture flasks and microtiter plates, were purchased from Lasec SA (Pty) Ltd. (Midrand, South Africa).

### 2.2 Plant material collection and extraction

Fresh plant material of *A. schoenoprasum*, *A.* × *proliferum* were collected in February 2024, and *S. aurea* in March 2021 from the Manie van der Schijff Botanical Garden (−25.752406, 28.229718) at the University of Pretoria. Fresh plant material of *Eriocephalus africanus*, *C. amboinicus*, and *Pelargonium graveolens* were collected from Future Africa Campus (−25.749741, 28.261216) in February 2024 at the University of Pretoria. The fresh plant material was cut into smaller pieces, and added to four parts absolute ethanol (99%) (4 mL ethanol to 1 g plant material) and sonicated for 15 minutes, and subsequently shaken for 48 h. This process was repeated 3–4 times until the extracting ethanol ran clear. The plant material was extracted at room temperature (25 °C), and then sonicated for 30 min, and subsequently placed on a shaker for 48 h before being vacuum-filtered and freeze-dried to obtain ethanolic plant extracts. The extracts were then stored at 4 °C until further use. The extraction yields of each plant are displayed in Table 2. The extracts were referred to by the following abbreviations: *Allium schoenoprasum* L (AS), *Allium* × *proliferum* (Moench) Schrad. Ex Willd (AX), *Coleus amboinicus* Lour (CA), *E. africanus* L (EA), *P. graveolens* L’Hér (PG), and *Salvia aurea* L (SA).

**TABLE 2:** Percentage extraction yield of the candidate aromatic plants.

Species name	Pru number	Fresh mass (g)	Amount of ethanol used (mL)	Extract mass (g)	Percentage yield (%)
*Allium schoenoprasum* L	0,132,961	265	1,060	4.7942	1.81
*Allium* *×* *proliferum* (Moench) Schrad. Ex Willd	0,132,962	480	1920	7.3667	1.53
*Coleus amboinicus* Lour	0,132,963	475	1900	7.2741	1.53
*Eriocephalus africanus* L	0,132,964	335	1,340	9.3238	2.78
*Pelargonium graveolens* L’Hér	0,132,965	175	700	1.5415	0.88
*Salvia aurea* L	128,685	250	1,000	5.512	2.20

### 2.3 Antibacterial assay

The protocol was followed according to the methods described by Munive Nuñez et al. (2024) with modifications (Munive Nuñez et al., 2024). A pure culture of *S. aureus* (ATCC 25923) was maintained on TS agar plates in aerobic conditions and subcultured every 24 h. The plant extracts were evaluated for their antibacterial activity according to the method described by Lall et al. (2013). Briefly, *S. aureus* subcultures were inoculated in correspondence with the 0.5 McFarland standard (1.5 x 10^8^ colony forming units per mL (CFU/mL)) in broth. The resulting solutions were diluted further for use in the assay (1:50,000). Stock concentrations of the extracts were prepared (4 mg/mL in 10% DMSO) and serially diluted to test at a final concentration of 7.81–1,000 μg/mL. The positive control, vancomycin was prepared at a stock concentration of 200 μg/mL and tested at a final concentration of 0.781–100 μg/mL. Additional controls included broth without bacteria (which served as the 0% viability control), broth with bacteria (which served as the 100% viability control) and a solvent vehicle control (dimethyl sulfoxide (DMSO), tested at a final concentration of 0.02%–2.5% v/v). Bacteria was added to all wells (excluding the broth only controls) and the plates were incubated at 37 °C for 16 h. Subsequently, 20 µL of PrestoBlue® cell viability reagent was added to all wells to act as an indicator for bacterial growth, where living cells maintain a reducing environment within their cytoplasm and mitochondria, and the blue resazurin salt of PrestoBlue® is reduced to a pink resorufin salt by viable cells. The minimum inhibitory concentration (MIC) was then visually determined based on the observed colour change, and the half maximal inhibitory concentration (IC_50_) was determined using the fluorescence readings which were measured with a VICTOR_ Nivo™ Multimode Microplate Reader (PerkinElmer South Africa (Pty) Ltd.), at an excitation of 560 nm and an emission of 590 nm. The following formula was used to determine the IC_50_ of the plant extracts against *S. aureus* ATCC 25923:

$$\%\text{ Bacteria viability}=\left[\frac{\left(A_{\text{treatment}}-A_{\text{blank}}\right)}{\left(A_{\text{control}}-A_{\text{blank}}\right)}\right]\times 100\%$$

The minimum bactericidal concentrations (MBCs) were determined by aliquoting 100 µL of bacterial culture from the wells exceeding the MIC value, and the well below the MIC as a positive control, onto TS agar plates and incubated for 24 h at 37 °C, after which bacterial growth was assessed and the MBC determined by observing no visible colony formation on the plate (Liu et al., 2024).

### 2.4 Cell culture

Human keratinocytes (HaCaT) were cultured in DMEM supplemented with 10% heat inactivated FBS, 1% amphotericin B (250 μg/mL), and 1% antibiotics (100 μg/mL streptomycin and 100 U/mL penicillin) and were grown under incubation at 37 °C and 5% CO_2_. After an approximately 80% confluent monolayer formed at the bottom of the flask, cells were subcultured using 0.25% trypsin-0.01% EDTA for 5 min and subsequently resuspended in DMEM media.

#### 2.4.1 Antiproliferative effects on HaCaT cells

The antiproliferative activity of the extracts were tested using HaCaT cells according to the method described by Lall et al. (2013). Cells were plated in 96-well microtitre plates at a concentration of 5 x 10^4^ cells/mL (5,000 cells per well) and allowed to attach at 37 °C for 24 h in an incubator set at 5% CO_2_. Actinomycin D was used as a positive drug control and tested at a final concentration of 0.00156–0.05 μg/mL. Other controls included media with (100% cell viability) and without cells (0% cell viability), a vehicle control (0.5% DMSO) and a toxic inducer control (DMSO tested at a final concentration of 0.625%–20%). The extracts (at stock concentrations of 100 mg/mL in DMSO) were serially diluted and tested at a final concentration of 31.25–1,000 μg/mL. The plates were incubated further for 72 h, and subsequently 20 µL of PrestoBlue®_ reagent was added to all wells (to act as an indicator for cell viability) and the plates were incubated for an additional 2 h to allow a colour change to occur. The fluorescence of the wells were then measured at an excitation of 560 nm and an emission of 590 nm using a VICTOR_ Nivo™ Multimode Microplate Reader (PerkinElmer South Africa (Pty) Ltd.). Percentage cell viability was calculated for each test well using the following formula:

$$\%\text{ Cell viability}=\left[\frac{\left(A_{\text{treatment}}-A_{\text{blank}}\right)}{\left(A_{\text{control}}-A_{\text{blank}}\right)}\right]\times 100\%$$

With ‘A’ indicating the fluorescence, ‘A_blank_’ indicating fluorescence of media without cells (0% cell viability control), ‘A_control_’ indicating the fluorescence of the vehicle control (1% DMSO) and ‘A_treatment_’ indicating the fluorescence of the treatment. Analyses were performed using the GraphPad Prism Version 4.0 (San Diego, California, United States of America) software to obtain the IC_50_ values of the extracts and controls (Lall et al., 2013).

The selectivity index (SI) for each extract was determined using the following formula:

$$\text{SI value}=\frac{{\text{IC}}_{50}\;\text{against}\;\text{HaCaT}\;\text{cells}}{\text{MIC}\;\text{against}\;S.\;aureus\;\text{ATCC}\;25923}$$

#### 2.4.2 Scratch assay on HaCaT cells

The wound healing potential of the extracts were tested *in vitro* on HaCaT cells using MuviCyte™ imaging technology and software. The HaCaT cells were seeded at a concentration of 5.5 × 10^5^ cells/mL (55,000 cells/well) in 96-well micro-titre plates and incubated for 24 h at 37 °C and 5% CO2 to form a confluent monolayer. A scratch was made in the cell monolayer to simulate a wound, using the MuviCyte™ 96-well plate Scratcher and the media was replaced with new media supplemented with or without sample treatment to remove cell debris. The extracts were tested at two non-toxic concentrations (25 and 50 μg/mL) as determined from the antiproliferative assay. An untreated media control and solvent vehicle control (0.25% DMSO) was also tested. The plate was placed in the MuviCyte Live-Cell Imaging System (PerkinElmer/Revvity South Africa (Pty) Ltd.) for 18 h. Percentage wound closure was calculated for each test well using the following formula:

$$\%\text{ Wound closure}=\left[\frac{\left(A_{0h}-A_{18h}\right)}{\left(A_{0h}\;\right)}\right]\times 100\%$$

With ‘A’ representing area of scratch at specific time points.

After the images were taken, a cell viability test was conducted on the plate.

### 2.5 Gas chromatography mass spectrometry

The method was adapted from Akanni et al. (2018) and was conducted by the Department of Chemistry (University of Pretoria). Briefly, compound separation and identification were done by an Agilent 7890A GC coupled to an Agilent 5975C inert MSD (Chemetrix (Pty) Ltd., Johannesburg, South Africa). The carrier gas, helium, was of ultra-high purity (UHP) grade (Afrox, South Africa) and was set at a flow rate of 1.0 mL/min in the constant flow mode. Extracts were resuspended in GC-MS graded methanol (99.9%) and injected at a volume of 1µL into a GC capillary column at 250 °C (Rxi-5 SilMS 30m × 0.25mm ID x 0.25 µm film thickness (Restek, Bellefonte, PA, United States of America). The GC inlet was operated in the splitless mode with a splitless time of 30s. The GC primary oven temperature programme was 40 °C (3min) at 10 °C/min to 300 °C (5min). For a total run time equal to 34min. The MS transfer line temperature was set at 280 °C and the ion source temperature at 230 °C. The electron energy was 70 eV in the electron impact ionisation mode (EI+), the mass acquisition range was 40–450 Da in full scan mode, and the detector voltage was set at 1750 V. Identification of compounds was done by comparison of mass spectral to that of the Wiley library (Wiley/NIST Mass Spectral Library) and compounds reported had a spectral match quality ≥80% (Akanni et al., 2018).

### 2.6 Statistical analysis

Antibacterial, antiproliferative and wound healing assays were performed in three independent experiments, with each sample tested in triplicate within each experiment (*n* = 3). Technical replicates were averaged for each independent experiment, and the final IC_50_ and percentage wound closure results were expressed as the mean ± standard deviation of the three independent experiments. For antibacterial and antiproliferative assays, cell viability was assessed using PrestoBlue® viability reagent fluorescent readings, and the obtained fluorescent data were normalized relative to 0% and 100% cell viability controls and used to generate sigmoidal dose-response curves to obtain the antibacterial IC_50_ values, calculated using non-linear regression analysis in GraphPad Prism version 4.0, with respective bottom and top constraints of 0% and 100%. Antibacterial MIC were determined from the fluorescence-based viability results and was also confirmed visually as the lowest concentration that showed no visible colour change, and MBC was defined as the lowest concentration that resulted in no visible colony growth. For the wound healing assays, statistical analysis was performed using one-way analysis of variance (ANOVA), followed by Dunnett’s multiple comparison test in GraphPad Prism version 4.0. Treated samples, including the untreated control, were compared to the vehicle control (0.25% DMSO solvent control), and p-values were reported where applicable, where *p < 0.05 and **p < 0.01 indicate statistical significance.

## 3 Results

### 3.1 Antibacterial activity against breast mastitis-associated bacteria

The MIC and MBC analysis identified that the ethanolic plant extracts of *C. amboinicus*, *E. africanus*, *P. graveolens*, and *S. aurea* displayed antibacterial activity against strains of *S. aureus.* The MIC and MBC values are displayed in Table 3 alongside their ratio (MBC:MIC). All other extracts displayed no antibacterial activity against *S. aureus* ATCC 25923 at the highest tested concentrations (MIC >1,000 μg/mL). The vancomycin positive control against *S. aureus* exhibited an MIC of approximately 3.125 μg/mL. There are no previous studies reporting the anti-bacterial activity of *S. aurea* and *A. × proliferum* on *S. aureus* and *C. amboinicus* has only been evaluated previously using disc diffusion. The MIC and MBC were visually determined and corresponds to the highest concentration where the added PrestoBlue®_ reagent is blue in colour and no bacterial colony growth observed on TS agar plates after 24 h, respectively (Soothill et al., 1992).

**TABLE 3:** The minimum inhibitory concentrations (MIC), minimum bactericidal concentration (MBC), the ratio MBC/MIC, and half-maximal inhibitory concentration (IC_50_) against *Staphylococcus aureus* (ATCC 25923) of the ethanolic extracts of six aromatic plant species and the positive drug control vancomycin.

Aromatic plant	MIC against *Staphylococcus aureus* (ATCC 25923) (μg/mL)	MBC against *Staphylococcus aureus* (ATCC 25923) (µg/mL)	MBC/MIC against *Staphylococcus aureus* (ATCC 25923)	IC_50_ against *Staphylococcus aureus* (ATCC 25923) (μg/mL) (IC_50_ ± SD)
*Allium schoenoprasum* L	>1,000	-	ND^1^	>1,000
*Allium* *×* *proliferum* (Moench) Schrad. Ex Willd	>1,000	-	ND	>1,000
*Coleus amboinicus* Lour	250	>1,000	ND	253.85 ± 16.68
*Eriocephalus africanus* L	250	1,000	4	252.66 ± 16.87
*Pelargonium graveolens* L’Hér	250	>1,000	ND	240.75 ± 12.94
*Salvia aurea* L	15.625	250	16	8.09 ± 0.77
Vancomycin (+)	3.125	6.25	2	1.11 ± 0.08

^1^ND, not determined.

### 3.2 Antiproliferative activity on HaCaT cells

The antiproliferative effects of the extracts on HaCaT cell viability was tested to determine whether the extracts were potentially toxic against normal human skin cells. The results would further compare whether the non-toxic concentrations correspond to the displayed antibacterial activity. The calculated IC_50_ value for *S. aurea* was determined to be 75.80 ± 4.58 μg/mL, while all other plant candidates had a calculated IC_50_ value greater than the highest tested concentration (>1,000 μg/mL). The positive control Actinomycin D and DMSO as a toxic inducer had the calculated IC_50_ values of 0.2338 ± 0.013 μg/mL and 6.58% ± 0.25%, respectively. The selectivity indices were determined using the MIC and IC_50_ values for each plant extract (Table 4).

**TABLE 4:** The minimum inhibitory concentrations (MIC) against *Staphylococcus aureus* (ATCC 25923) and the half-maximal inhibitory concentration (IC_50_) against human keratinocytes (HaCaT) of the tested aromatic plants and their selectivity index (SI), an SI > 1 shows the aromatic plant extract favorably inhibits bacteria without cytotoxic damage to HaCaT cells.

Aromatic plant	MIC against *Staphylococcus aureus* (ATCC 25923) (μg/mL)	IC_50_ against human keratinocytes (HaCaT) (IC_50_ ± SD) (μg/mL)	Selectivity index
*Allium schoenoprasum* L	>1,000	>1,000	ND
*Allium* *×* *proliferum* (Moench) Schrad. Ex Willd	>1,000	>1,000	ND
*Coleus amboinicus* Lour	250	>1,000	>1
*Eriocephalus africanus* L	250	>1,000	>1
*Pelargonium graveolens* L’Hér	250	>1,000	>1
*Salvia aurea* L	15.625	75.80 ± 4.58	4.85

### 3.3 Wound healing effect on HaCaT cells

**FIGURE 1:**
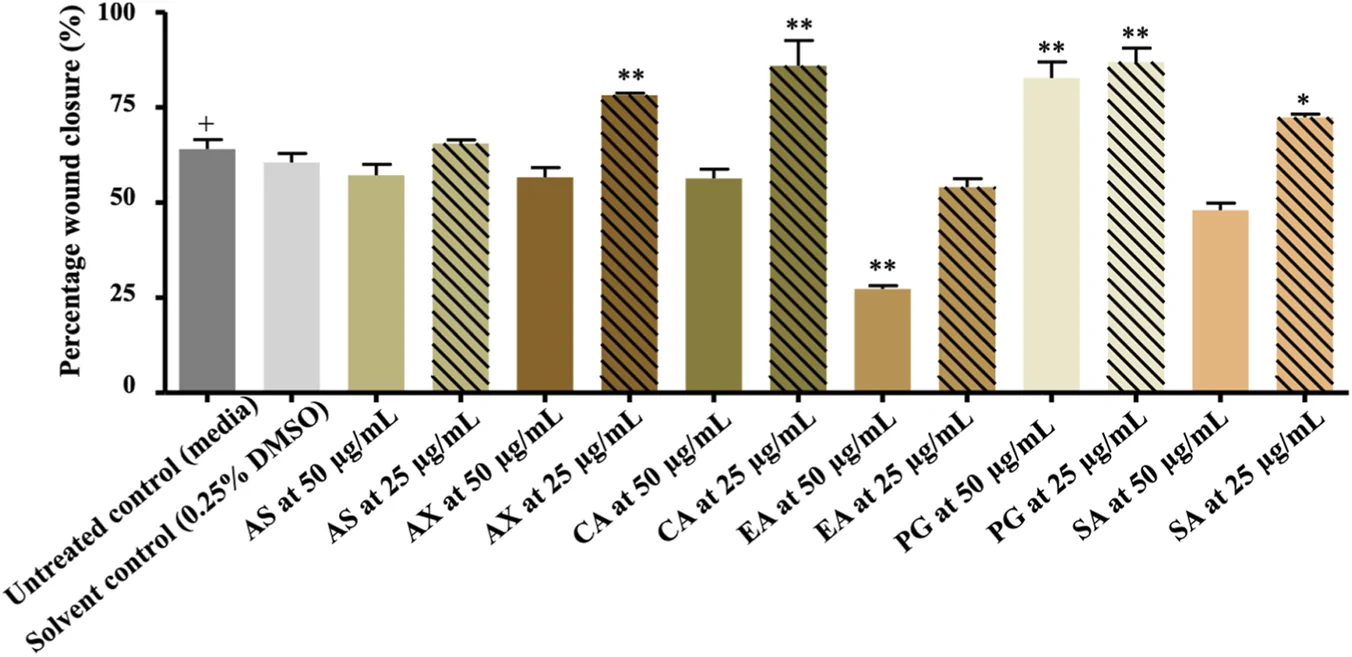
Percentage wound closure (%) of the ethanolic extracts of AS, AX, CA, EA, PG, and SA at 25 and 50 mg/mL on HaCaT cells after 18 h. Values are displayed as mean ± standard deviation where *p < 0.05 and **p < 0.01 indicate statistical significance (one-way ANOVA and Dunnett’s multiple comparison test) when compared to 0.25% (vehicle) DMSO control (+).

The extracts were evaluated for wound healing effect on HaCaT cells. The percent closure was taken at 0 h and 18 h for evaluation. The percentage wound closure is indicated in Figure 1. The solvent had no effect on wound closure as there was no statistically significant difference (p > 0.05) determined between the vehicle control (60.52% ± 4.65%) to the untreated media control (63.99% ± 4.97%).

The extracts which showed significant wound healing activity (*p* < 0.05) were *S. aurea* (at 25 μg/mL) with a percentage wound closure of 72.31% ± 1.48%. *Eriocephalus africanus* (at 50 μg/mL) showed significant wound healing inhibition (*p* < 0.05) with a percentage wound closure of 27.26% ± 1.48%. Three ethanolic leaf extracts, namely, *A. × proliferum* (at 25 μg/mL)*, C. amboinicus* (at 25 μg/mL), and *P. graveolens* (at 50 μg/mL) showed the more significant wound healing activity (p < 0.01) with percentage wound closures of 78.15% ± 0.98%, _79.47% ± 2.83%, and 82.75% ± 7.26% respectively. With *P. graveolens* (at 25 μg/mL) having the best wound healing activity (*p* < 0.01) with a percentage wound closure of 86.94% ± 6.21%.

### 3.4 Gas-chromatography mass spectrometry of selected plant extracts

**FIGURE 2:**
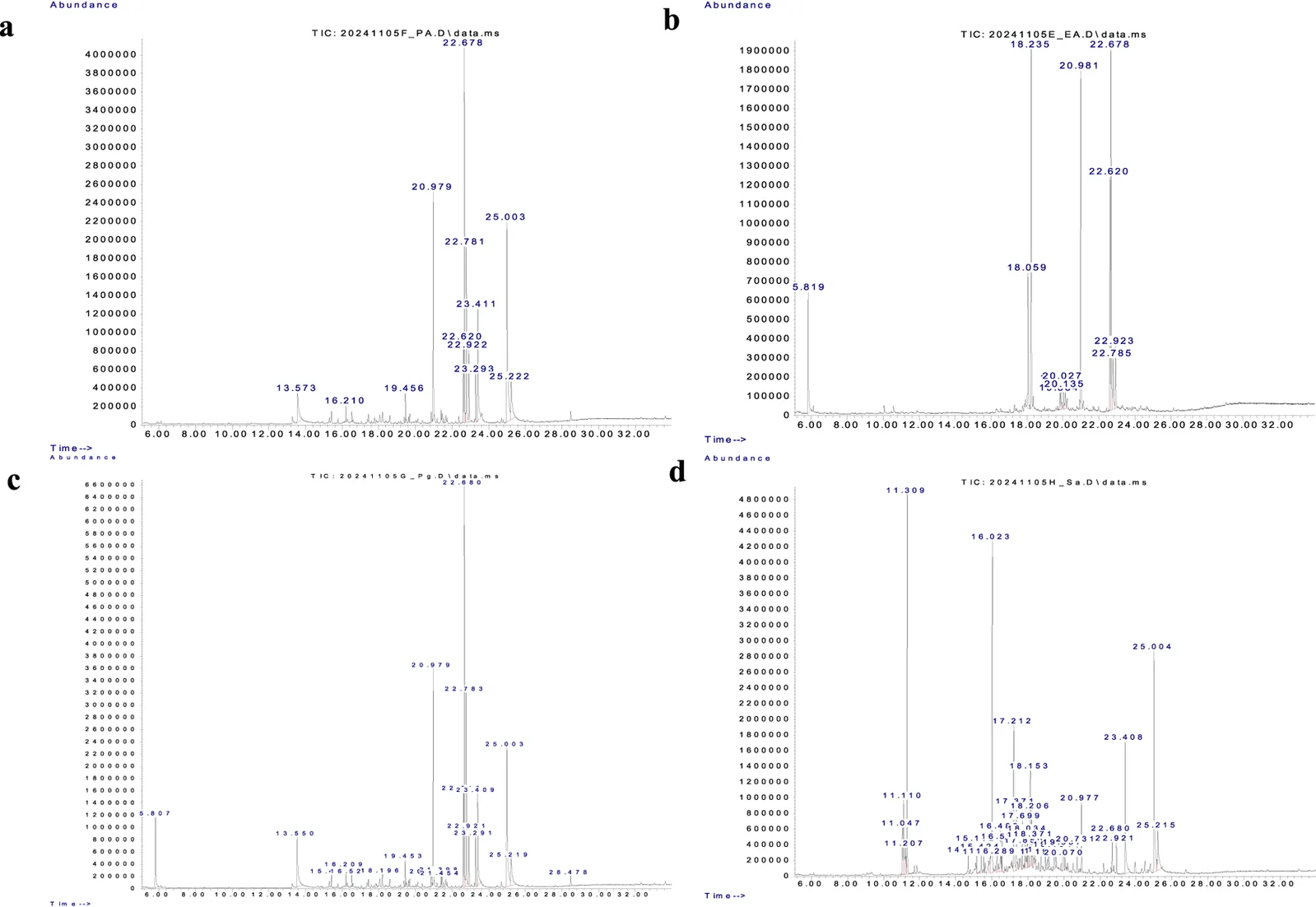
The GC-MS chromatograms of ethanolic fresh plant material extracts of **(a)** CA, **(b)** EA **(c)** PG, and **(d)** SA.

**FIGURE 3:**
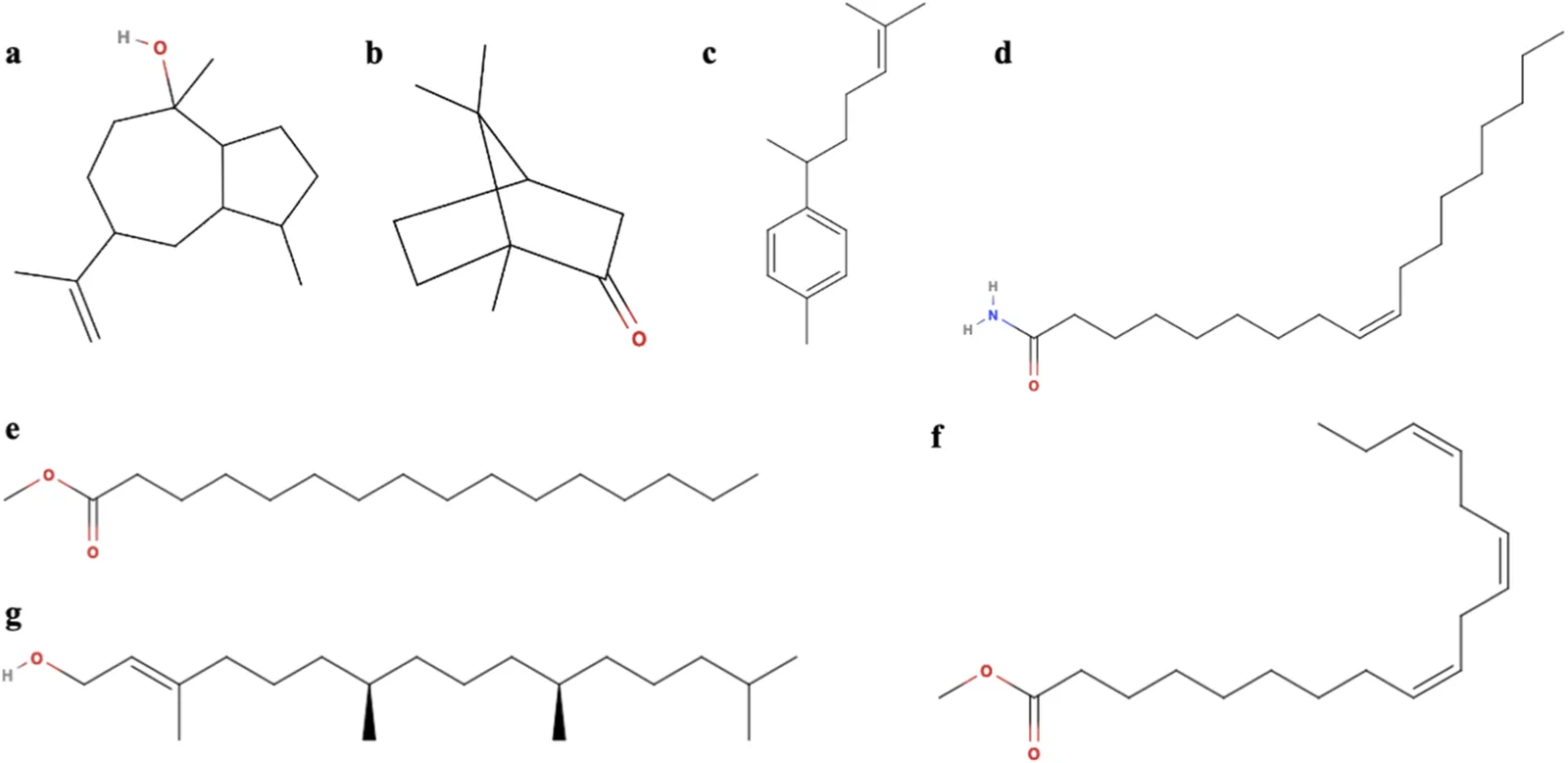
Compounds with greater than 10% abundance. **(a)** Pogostol isolated from EA **(b)** camphor isolated from SA **(c)** a-curcumene isolated from SA **(d)** oleamide isolated in CA, PG, and SA **(e)** methyl palmitate isolated from CA, EA, PG, and SA **(f)** methyl linolenate isolated from CA, EA, and PG and **(f)** phytol isolated from CA, EA, and PG. Generated using MolView 2026.

Due to their displayed antibacterial activity at the tested concentration range (IC_50_ < 1,000 μg/mL), and plants which selectively inhibited the anti-bacterial growth compared to its cytotoxic effect on HaCaT (SI > 1), *C. amboinicus*, *E. africanus*, *P. graveolens* and *S. aurea* were selected to undergo GC-MS analysis to identify present compounds. A total of 29 volatile compounds were identified across the ethanolic extracts of *C. amboinicus*, *E. africanus*, *P. graveolens*, and *S. aurea*. The chromatograms are compiled in Figure 2. The compounds with the highest spectral match qualities (%) are compiled in Table 4. Figure 3 shows the compounds isolated with an abundance (%) greater than 10. The bioactive compound camphor identified in *S. aurea* (20.45% abundance with a 98% spectral match quality) which displayed the best antibacterial activity. Oleamide identified in both *P. graveolens* and *C. amboinicus,* with an abundance of 27.73% and 18.80% respectively (99% spectral match quality), both extracts displayed significant wound healing activity Table 5.

**TABLE 5:** GC-MS spectral analysis of the compounds identified from each of the four selected ethanolic extracts CA, EA, PG, and SA.

Aromatic plant species	Retention time (min)	Abundance (%)	Formula	Molecular weight (g/mol)	Compound name	Aromatic plant(s) where compound also appeared and their abundance (%)	Spectral match quality range (%)
*Eriocephalus africanus* L	18.235	19.43	C_15_H_26_O	222.36	pogostol	-	95
	19.835	1.83	C_15_H_24_O	220.18	longifolenaldehyde	-	86
*Pelargonium graveolens* L’Hér	15.425	0.609	C_15_H_24_	204.35	α-zingiberene	-	91
	28.478	0.646	C_30_H_50_	410.73	squalene	-	98
	16.211	0.62	C_15_H_24_	204.35	δ-cadinene	*S. aurea* (1.55)	84–99
	18.197	1.16	C_15_H_26_O	222.37	α-cadinol	*E. africanus* (7.29)	94–98
*Salvia aurea* L	11.169	20.45	C_10_H_16_O	152.23	camphor	-	98
	14.697	0.871	C_15_H_24_	204.36	copaene	-	99
	15.163	2.128	C_15_H_24_	204.35	α-bergamotene	-	99
	15.559	0.546	C_15_H_24_	204.35	aromandendrene	-	99
	15.971	14.15	C_15_H_22_	202.17	α-curcumene	-	99
	16.287	0.953	C_15_H_24_	204.19	α-muurolene	-	97
	16.482	1.977	C_15_H_24_	204.35	α-amorphene	-	98
	16.578	1.197	C_15_H_22_	202.17	calamenene	-	96
	17.373	2.550	C_15_H_24_O	220.18	caryophyllene oxide	-	96
	17.701	1.656	C_15_H_24_O	220.18	humulene epoxide II	-	81
	20.730	1.411	C_15_H_26_O_2_	238.19	clovane-2,9-diol	-	98
	22.682	1.322	C_19_H_36_O_2_	296.72	methyl oleate	-	99
	23.406	6.888	C_14_H_29_NO	227.23	myristic amide	-	90
*Coleus amboinicus* Lour	13.573	5.08	C_10_H_14_O	150.10	carvacrol	*P. graveolens* (6.63)	94–95
	16.211	0.96	C_15_H_24_	204.19	β-selinene	*P. graveolens* (0.93)	99
	23.292	2.92	C_20_H_34_O_2_	306.26	ethyl linolenate	*P. graveolens* (2.84)	99
	23.411	9.63	C_16_H_33_NO	255.26	palmitamide	*P. graveolens* (7.26)	95–97
	20.978	11.56	C_17_H_34_O_2_	270.45	methyl palmitate	*E. africanus* (17.45), *P. graveolens* (11.12), *S. aurea* (2.57)	98–99
	22.621	4.37	C_19_H_34_O_2_	294.47	methyl linoleate	*E. africanus* (11.59) and *P. graveolens* (5.05)	99
	22.678	21.63	C_19_H_32_O_2_	292.46	methyl linolenate	*E. africanus* (21.93) and *P. graveolens* (22.83)	99
	22.782	10.45	C_20_H_40_O	296.31	phytol	*E. africanus* (4.11) and *P. graveolens* (10.87)	91–98
	22.921	4.14	C_19_H_38_O_2_	298.50	methyl stearate	*E. africanus* (3.93), *P. graveolens* (3.28), *S. aurea* (1.11)	98–99
	25.002	27.73	C_18_H_35_NO	281.48	oleamide	*P. graveolens* (18.80) and *S. aurea* (11.85)	99

## 4 Discussion

The extracts tested showed varying bioactivity and compound profile. A review by Zouine et al. (2024) defines a plant extract having significant antibacterial activity when the MIC value is less than 100 μg/mL, moderate activity between 100 and 625 μg/mL, and low activity at above 625 μg/mL. Thus, by applying these definitions, the tested extracts showed that *S. aurea* (15.625 μg/mL) was significantly active, whereas *C. amboinicus* (250 μg/mL)*, E. africanus* (250 μg/mL)*,* and *P. graveolens* (250 μg/mL) were moderately active (Zouine et al., 2024). *Allium schoenoprasum* and *A.* × *proliferum* showed low antibacterial activity (MIC >1,000 μg/mL) suggesting poorer activity than the aforementioned extracts (Tamokou et al., 2017). The positive control vancomycin had a much lower MIC of 3.125 μg/mL when compared to the plant extracts. This is expected as vancomycin is a pure compound which is highly potent while crude plant extracts possess a diverse range of compounds in minimal concentrations (Nasim et al., 2022). Therefore, crude extracts with significant activity can be taken for compound identification and isolation to determine which compound is responsible for the bioactivity and whether the compound is as potent as the drug control when tested at the same concentration. *Salvia aurea* shows promising significant antibacterial potential, and further investigation into its mechanistic action may give insight to novel antibacterial agents which may be beneficial for mastitis treatment. This could be done by testing a mutant strain of *S. aureus* such as *S. aureus* (ATCC 6538) against a wild-type strain such as *S. aureus* (ATCC 25923). If growth is inhibited in both the mutant and wild-type strains, an essential cellular process such as the maintenance of the cell wall, the central metabolism and respiration or another process required by the bacteria for survival, is blocked (Choueiry et al., 2022; Nikolic and Mudgil, 2023). This allows for the extract to target a wider range of *S. aureus* strains and suggests it could be used as an alternative inhibitor of bacterial growth against *S. aureus* (Campbell, 2011). In a study by Makade et al. (2024), an MBC:MIC ratio of ≤4 is considered to have a bactericidal effect, and a ratio of 4–32 has a bacteriostatic effect. *Salvia aurea* was determined to have an MBC:MIC ratio of 16 thus having a bacteriostatic effect. Bacteriostatic antimicrobials inhibit bacteria growth without bacterial death by targeting bacterial protein synthesis, such as inhibiting bacterial ribosomal subunits, resulting in preventing bacterial defence and allows for the host’s immune cells to eliminate offending bacteria (Loree and Lappin, 2023). Thus, further assays measuring concentrations of certain bacterial proteins such as folic acid can be used to test bacterial growth inhibition, this is especially relevant as *Lactobacilli* should remain intact for healthier breast milk (James and Martinez, 2008; Fernández et al., 2014). *Lactobacilli* are essential in healthy milk production and for increased infant health, it is thus important that the plant extracts are non-toxic to these bacteria. Broth microdilution assays are an effective way of testing for bacterial inhibition; thus, it is proposed that a future study can determine whether the extracts also inhibit *Lactobacilli* (Łubiech and Twarużek, 2020). *Eriocephalus africanus* had an MBC:MIC ratio of 4, indicating that it has a bactericidal effect (Makade et al., 2024). Bactericidal antimicrobials disrupt an essential cellular function of the bacteria, the positive control vancomycin (MBC:MIC ratio of 2) is a bactericidal antibiotic which inhibits bacteria cell wall biosynthesis (Ranjan Panda et al., 2025). With an increasing prevalence vancomycin-resistant *S. aureus*, further investigation of *E. africanus* can be conducted to determine the presence of novel bactericidal compounds (Lulitanond et al., 2026). This can be done using bioassay guided fractionation with a variety of solvents to determine the presence of bioactive compounds which may be responsible for the bactericidal activity displayed. This could also potentially help isolate compounds that are both antibacterial and non-toxic from the compounds that show toxicity but minimal to poor antibacterial activity. Varying solvent polarities can allow for compounds of all different polarities to be extracted from the plant, for example, non-polar solvents such as fatty acids will be more readily extracted in a non-polar solvent such as hexane while polar compounds are more readily extracted in polar solvents such as acetone (Akowuah et al., 2005). Compounds not isolated in the ethanolic extracts could be extracted using a different solvent, this could discover whether extracts may have stronger antibacterial activity than the ethanolic extracts and further expand the chemical profile of these plants leading to further testing of the plants’ *in vitro* bioactivity. Compounds such as camphor identified from *S. aurea* and carvacrol identified from both *C. amboinicus* and *P. graveolens*, have been shown in literature to have effective antibacterial activity, having displayed an MIC value of 15 μg/mL, and research has shown the ability of these compounds to rupture the bacterial cell membrane, camphor and carvacrol are lipophilic, as they are a terpenoid and phenolic monoterpene respectively, which easily permeates and disorders the bacteria’s lipid bilayer (Álvarez-Martínez et al., 2021; Abdollahi et al., 2024; Gan et al., 2025). The GC-MS results indicate a potential high abundance of camphor (20.45% in *S. aurea)* which could correlate to the *in vitro* bioactivity of the extract as it was classified to be significantly active against *S. aureus* (15.625 μg/mL) which suggests that these compounds could potentially contribute to the biological activity observed (Álvarez-Martínez et al., 2021). *Eriocephalus africanus* and *C. amboinicus* have displayed much better activity against *S*. *aureus* than previously reported (Table 1) which could be due to testing against a different strain of *S. aureus* or using a broth microdilution assay rather than disc diffusion, respectively. The scent produced by aromatic medicinal plants can be attributed to the volatile compounds which are especially concentrated in essential oils (EOs), the scent of a plant has been shown to be deeply correlated with a plant’s bioactivity (Mohammed et al., 2024). The EO from *P. graveolens* has been reported to have antibacterial activity and would make for an interesting comparison to the ethanolic extract in terms of MIC values (Ghannadi et al., 2012). Alternate treatments, such as plant extracts and their essential oils have great potential to act as a multi-facetted alternate treatment due to their large range of secondary metabolites which can work synergistically to inhibit bacterial growth and stimulate wound healing (Seow et al., 2014; Cowan, 1999). When mastitis is left untreated, painful breast abscesses can form which prevent the mother from breastfeeding. A study by Prakash et. a (2025) found that 79% of bacterial cultures successfully grown from pus samples taken from patients with lactational breast mastitis were *S. aureus* indicating the prevalence of *S. aureus* in these abscesses and the importance of investigating a treatment which can encourage wound healing after the removal of abscesses as well as inhibiting the spread of infection (Prakash et al., 2025). *Pelargonium graveolens* exhibited antibacterial activity on *S. aureus* (250 μg/mL) and showed statistically significant *in vitro* wound healing activity on HaCaT (at tested concentrations of 25 and 50 μg/mL with a percentage wound closure of 86.94% ± 6.21% and 82.75% ± 7.26% respectively (p < 0.01)). This *in vitro* activity warrants further exploration with mechanistic studies, and potentially evaluated with *in vivo* animal models. Furthermore, carvacrol (a compound identified from *P. graveolens*) has exhibited wound healing potential by supporting multiple states of wound repair including being anti-microbial, anti-inflammatory, and supporting tissue regeneration (Küpeli and Deliorman Orhan, 2025). Future studies can focus on determining whether the compounds identified using GC-MS do contribute to the antibacterial and wound-healing activity observed. Further isolating and confirming the presence of these compounds and assessing combinations of these compounds could show whether these compounds work synergistically, antagonistically or if the isolated compounds perform better than the crude ethanolic extracts. Milk stasis is a main causative of breast mastitis, and therefore a treatment that can help breakdown the excess breast milk can allow for a more effective treatment of mastitis. Probiotics, which encourage the growth of *Lactobacilli*, have indicated an effect on reducing the incidence of mastitis, and have shown to shorten healing time of single cavity lactational breast abscesses with no effect on safety during breastfeeding (Yu et al., 2022; Zhang et al., 2022). Thus, testing whether the extracts promote *Lactobacilli* production could indicate a potential probiotic effect which further encourages wound healing. Breast mastitis is a problem especially to mothers who solely rely on breastfeeding to sustain the infant; thus, it provides a wide scope of different avenues for an effective and reliable alternative to antibiotics and surgical treatment which is easily accessible and safe for both the mother and infant. Despite only *S. aurea* being found to have high antibacterial activity against *S. aureus* (ATCC 25923) in this study, it could be further evaluated by fractionation to determine the compounds responsible for the activity and determine whether these compounds could also be responsible for the statistically significant wound healing activity. Furthermore, *E. africanus* was found to have a bactericidal effect which could indicate the presence of bioactive compounds with the potential to prevent bacterial growth altogether. Breast mastitis requires a treatment which can rapidly clear infection and promote wound healing, this will allow for the continuation of breastfeeding while also avoiding further milk stasis which can result in painful abscesses. The extracts of *S*. *aurea*, alongside *A.* × *proliferum*, *C. amboinicus*, and *P. graveolens,* displayed inhibition of bacterial growth and significant stimulation of wound healing within less than a 24 h time period, indicating great potential for a rapid treatment. These results are indicative that these plant extracts can be further investigated through mechanistic studies and *in vivo* animal models or clinical trials to determine whether they could be a potential treatment which allows for a quicker recovery time so that the mother can return to breastfeeding without further delay. Furthermore, other plant-based alternatives could be investigated alongside their essential oil counterparts to determine the most effective line of treatment.

## 5 Conclusion

The ethanolic extracts of the chosen aromatic plants, *A. schoenoprasum, A.* × *proliferum, C. amboinicus, E. africanus, P. graveolens* and *S. aurea*, displayed varying degrees of *in vitro* antibacterial and wound healing activity. Among the tested extracts, *S. aurea* showed the most promising antibacterial activity, with an MBC:MIC of 16 indicating a bacteriostatic effect, whereas *E. africanus* displayed moderate antibacterial activity, with an MBC:MIC of four which also indicates a bactericidal effect. These findings indicate that these selected aromatic plants may contain bioactive compounds with potential relevance for the management of mastitis-associated bacterial infections. In addition to their antibacterial effects, several extracts demonstrated promising *in vitro* wound healing activity, where *A. schoenoprasum, A.* × *proliferum, C. amboinicus, E. africanus, P. graveolens* and *S. aurea* significantly enhanced wound closure in comparison to the untreated control (p < 0.05), supporting their potential role in promoting tissue repair. In order to additionally assess these plants for their suitability for future breast mastitis research, further anti-inflammatory activities may be evaluated, which is further supported by the identified compounds from the GC-MS data (Table 4) which are known to possess anti-inflammatory mechanisms. In contrast, *E. africanus* inhibited wound closure by 27.26% ± 1.48%, which may suggest a possible antimigratory effect, therefore potentially warranting further investigation in cancer-related models whereby inhibition of cell migration is therapeutically relevant. Overall, *S. aurea* emerged as the most promising candidate due to the notable antibacterial activity and significant stimulation of wound closure, however, the extract showed to be the most toxicity towards HaCaT cells, albeit the extract still showed selectivity towards the pathogen *S. aureus* in comparison. Therefore, bioassay-guided fractionation studies are recommended to isolate and identify the active antibacterial and wound healing promoting compounds, which may preferably exhibit less cytotoxicity, present within the extract. Based on the GC-MS data collected on the sample, camphor was identified as the potential compound which may be responsible for the observed activity, although targeted compound testing should be conducted to confirm this.

Further studies can include the extracts undergoing liquid chromatography-mass spectroscopy (LC-MS) for comparison to determine whether any non-volatile compounds exist at higher concentration that potentially contribute to the observed bioactivity. This study showed that medicinal aromatic plants possess promising *in vitro* antibacterial and wound-healing properties relevant to breast mastitis management, future studies can focus on isolating compounds from *C. amboinicus, E. africanus, P. graveolens* and *S. aurea* as these extracts showed promise for future mastitis research.

## Data Availability

The raw data supporting the conclusions of this article will be made available by the authors, without undue reservation.
